# Clinical Utility of the Cardiorespiratory Optimal Point in Patients with Heart Failure

**DOI:** 10.1249/MSS.0000000000003206

**Published:** 2023-05-13

**Authors:** SOPHIE H. KROESEN, ESMÉE A. BAKKER, JOHAN A. SNOEK, ROLAND R. J. VAN KIMMENADE, JEROEN MOLINGER, CLAUDIO G. ARAÚJO, MARIA T. E. HOPMAN, THIJS M. H. EIJSVOGELS

**Affiliations:** 1 Department of Physiology, Radboud Institute for Health Sciences, Radboud university medical center, Nijmegen, THE NETHERLANDS; 2 Department of Physical Education and Sports, Sport and Health University Research Institute (iMUDS), University of Granada, Granada, SPAIN; 3 Isala Heart Center, Zwolle, THE NETHERLANDS; 4 Sports Medicine Department Isala, Zwolle, THE NETHERLANDS; 5 Department of Cardiology, Radboud Institute for Health Sciences, Radboud university medical center, Nijmegen, THE NETHERLANDS; 6 Duke Human Pharmacology and Physiology Lab (HPPL), Department of Anesthesiology, Duke University Medical Center, Durham, NC; 7 Exercise Medicine Clinic (CLINIMEX), Rio de Janeiro, BRAZIL

**Keywords:** CARDIOPULMONARY EXERCISE TESTING, CARDIAC REHABILITATION, VENTILATORY EFFICIENCY, CARDIOVASCULAR RISK, PHYSICAL FITNESS

## Abstract

**Introduction:**

We assessed the cardiorespiratory optimal point (COP)—the minimal *V̇*_E_/V̇O_2_ in a given minute of an incremental cardiopulmonary exercise test—in patients with heart failure (HF) and aimed to determine 1) its association with patient and disease characteristics, 2) changes after an exercise-based cardiac rehabilitation program (CR), and 3) the association with clinical outcomes.

**Methods:**

We studied 277 HF patients (67 (58–74) yr, 30% female, 72% HF with restricted ejection fraction) between 2009 and 2018. Patients participated in a 12- to 24-wk CR program, and COP was assessed pre- and post-CR. Patient and disease characteristics and clinical outcomes (mortality and cardiovascular-related hospitalization) were extracted from patient files. The incidence of clinical outcomes was compared across COP tertiles (low, <26.0; moderate, 26.0–30.7; high, >30.7).

**Results:**

Median COP was 28.2 (24.9–32.1) and was reached at 51% ± 15% of V̇O_2peak_. Lower age, female sex, higher body mass index, the absence of a pacemaker or the absence of chronic obstructive pulmonary disease, and lower N-terminal prohormone brain natriuretic peptide concentrations were associated with a lower COP. Participation in CR reduced COP (−0.8; 95% confidence interval, −1.3 to −0.3). Low COP had a reduced risk (adjusted hazard ratio, 0.53; 95% confidence interval, 0.33–0.84) for adverse clinical outcomes as compared with high COP.

**Conclusions:**

Classic cardiovascular risk factors are associated with a higher, more unfavorable, COP. CR-based exercise training reduces COP, whereas a lower COP is associated with a better clinical prognosis. As COP can be established during a submaximal exercise test, this may offer novel risk stratification possibilities for HF care programs.

One in five adults develops heart failure (HF) during lifetime (1), and absolute numbers are rising given the aging population (2). HF is associated with a lower quality of life, lower cardiorespiratory fitness (CRF) (3), and a 50% risk of mortality within 5 yr after diagnosis (4). Assessing CRF by measuring V̇O_2peak_ in a maximal cardiopulmonary exercise test (CPET) is a powerful tool to acquire information about disease severity and prognosis and to guide clinical management strategies as recommended in international guidelines (Class 1-C) (5,6). Nevertheless, CRF assessment remains underutilized in clinical settings (5), as CPETs can be considered expensive, time-consuming, and burdensome for HF patients, and require specific skills and equipment.

The cardiorespiratory optimal point (COP) is a novel cardiopulmonary variable that can be derived from a submaximal exercise test with expired gas analysis (7) and is typically reached at 30%–50% of V̇O_2peak_ in healthy individuals (8). COP has been defined as the lowest value of the ventilatory equivalent for oxygen (ratio between ventilation (*V̇*_E_) and oxygen consumption (V̇O_2_)) in a given minute of CPET and does not require determining (anaerobic) threshold, which minimizes the interobserver variation (7). Cohort studies in the general population and community-dwelling adults showed that higher COP values are associated with a higher risk of sudden cardiac death (8), cardiovascular (CV) (9), and all-cause mortality (9,10). COP could therefore be an alternative submaximal variable to assess aerobic performance, but information about the association with patient or disease characteristics, the impact of exercise training, and its prognostic value in HF patients is currently limited in the literature.

Therefore, we assessed the COP in patients with HF and aimed to determine 1) its association with patient and disease characteristics, 2) changes after an exercise-based cardiac rehabilitation (CR) program, and 3) the association with adverse clinical outcomes. We hypothesized that COP will be 1) higher in HF patients with more CV risk factors, 2) improved after CR, and 3) inversely associated with the incidence of adverse relevant clinical outcomes during long-term follow-up.

## METHODS

### Study design and population

HF patients registered in the *HArtfalen Registratie Project* (HARP) database (11) and those who participated in the outpatient CR program at the Isala Clinic (Zwolle, the Netherlands) between October 2009 and January 2018 were eligible for this study. HF patients with a reduced ejection fraction (HFrEF), midrange ejection fraction, and preserved ejection fraction were included if they performed a CPET pre- and/or post-CR. Participants provided informed consent before registration in the HARP database. The study complies with the Declaration of Helsinki, and the local medical ethics committee approved the study protocol (no. 2021-13378).

### Patient and disease characteristics

Electronic patient files were used to collect 1) patient characteristics including age, sex, body mass index (BMI), smoking status, presence of diabetes mellitus, and chronic obstructive pulmonary disease (COPD), and 2) HF characteristics including left ventricular ejection fraction, type of HF, HF etiology (ischemic, idiopathic, valvular or other), New York Heart Association (NYHA) class, medical device implementation (implantable cardioverter–defibrillator, pacemaker, or cardiac resynchronization therapy), cardiac comorbidities (atrial tachycardia, atrial fibrillation, percutaneous transluminal coronary angioplasty, coronary artery bypass graft, or heart transplantation), medication use (angiotensin-converting enzyme inhibitor, angiotensin-receptor blocker, aldosterone receptor antagonist, β-blocker, diuretics, and statins), and laboratory values (N-terminal prohormone brain natriuretic peptide (NT-proBNP), modification of diet in renal disease, hemoglobin, and sodium).

### Cardiac rehabilitation

Patients participated in two distinct exercise-based CR programs at the Isala Clinic. Patients enrolled between 2009 and 2010 (6-month CR, *n* = 57) followed by a 3-month supervised graded exercise training program with 3 sessions per week at moderate exercise and including resistance training at the outpatient clinic. Patients were encouraged to train at home the other days. Thereafter, patients were stimulated to train 2 sessions per week under the guidance of a physiotherapist in their home-based environment for another 3 months (11). Patients enrolled between 2011 and 2018 (3-month CR, *n* = 220) also followed a 3-month supervised exercise training program with 2 sessions per week at a moderate exercise intensity at the outpatient clinic, but were recommended to continue their exercise regimen by themselves after completion of the CR program. Adherence to the CR program was assessed by the percentage of attended to the total number of exercise sessions in the program.

### Cardiopulmonary exercise test

A symptom-maximal CPET on a cycle ergometer (Lode Corrival, Lode, Groningen, the Netherlands) was performed at baseline (pre-CR) and at 6 months (post-CR). O_2_ and CO_2_ partial pressures were continuously sampled by a mass spectrometer (MetaMax IIIb; Cortex, Leipzig, Germany), which was calibrated before every test by both ambient air and a fixed known gas mixture. V̇O_2_, *V̇*_E_, and carbon dioxide production (V̇CO_2_) were computed by breath-by-breath analysis (12). Heart rate was measured continuously by electrocardiography, and lactate was determined at rest, before the start of the test, and immediately at its end. A personalized ramp protocol was planned aiming a CPET duration of 8–12 min. The workload was increased gradually until the participant reached exhaustion or was unable to maintain 60 rpm. All participants were verbally encouraged throughout the CPET to reach maximum.

### COP assessment

The COP, a dimensionless variable, was calculated by obtaining the lowest *V̇*_E_/V̇O_2_ value in a given minute (7) using the raw CPET data. COP was measured pre- and post-CR. Unless otherwise indicated, in this article, COP stands for the COP measured pre-CR. Change in COP was defined as the absolute numerical difference in COP from pre- to post-CR. We determined the V̇O_2_ at which the COP was reached and expressed it as a percentage of V̇O_2peak_. V̇O_2peak_, an indicator of CRF, was defined as the highest 30-s value that was reached during the exercise protocol and was adjusted for body weight (mL·kg^−1^·min^−1^). Peak heart rate (bpm), workload (W), *V̇*_E_ (mL·min^−1^), and respiratory exchange ratio were obtained in the CPET.

### Mortality and unplanned hospitalization

The survival status of study participants was assessed using the Dutch National Death Registry on June 21, 2022. Furthermore, the incidence of CV-related unplanned hospitalization due to HF, acute coronary syndrome, rhythm or conduction abnormalities, valvular abnormalities, infectious disease affecting the heart, and cerebrovascular accidents (transient ischemic attack or stroke) was extracted from the electronic patient files and assessed between CR enrollment (2009–2018) and April 11, 2022.

### Statistical analysis

All statistical tests were performed using R version 4.2.1 with packages “lme4, “survminer,” and “survival.” All tests were two-sided, and *P* < 0.05 was considered statistically significant. Continuous normally distributed data are presented as mean ± SD, continuous nonnormally distributed data as median (interquartile range (IQR)), and categorical variables as number (%). All data were visually inspected for normality, and the Shapiro–Wilk test was performed. A *χ*^2^ test was used to compare the adherence between 3- and 6-month CR. For the first aim of our study, univariable and multivariable linear regression analyses were used to assess the association between patient and disease characteristics and V̇O_2peak_ with COP and change in COP. Variables with a *P* value <0.10 were included in the multivariable linear regression model using backward selection. For the second aim, change in pre- to post-CR COP, other cardiopulmonary variables, and CV (cardiovascular disease (CVD)) risk factors were evaluated using linear mixed model analysis with random intercepts and time (pre- to post-CR) as categorical variable. The estimates were adjusted for sex, age, type of HF, and NYHA class. A sensitivity analysis was performed by repeating the linear mixed model analysis in HF patients with both a pre- and post-CR COP value (*n* = 200) and patients without COPD. Finally, the association between tertiles of COP (i.e., low COP: <26.0; moderate COP: 26.0–30.7; high COP: >30.7) and tertiles of changes in COP (i.e., improved COP: >1.0, stable COP: −2.1 – 1.0, deteriorated COP: <−2.1) with adverse clinical outcomes was assessed using Kaplan–Meier curves and the log-rank test. The age-adjusted hazard ratio (HR) with 95% confidence interval (95% CI) and multivariable-adjusted HRs for age, sex, type of HF, NYHA class, and COPD were estimated with univariable and multivariable Cox proportional hazard models. As a sensitivity analysis, V̇O_2peak_ was added to the multivariable-adjusted Cox proportional hazard models. Change in COP was additionally adjusted for pre-CR COP.

## RESULTS

### Participants

A total of 280 HF patients met our inclusion criteria, but raw CPET data were missing for 3 patients. Therefore, the analytical cohort consisted of 277 patients (Table 1). Participants were 67 (58–74) yr old, 30% was female, and the BMI was 29 ± 5 kg·m^−2^. Most participants had HFrEF (72%) and an NYHA class of 2 (52%) or 3 (37%). Pre-CR data were available in 267 patients and post-CR data in 210 patients, whereas 200 patients had pre- and post-CR data available (see Supplemental Fig. 1, Supplemental Digital Content, Flowchart of the study, http://links.lww.com/MSS/C862). The large majority (76%) of HF patients participated in >80% of CR sessions, and this was comparable between the 3- (74%) and 6-month CR programs (82%, *P* = 0.24).

**TABLE 1:** Patient and disease characteristics of the study cohort (*n* = 277) and the COP tertiles.

	Total Population (*n* = 277)	High COP (*n* = 89)	Moderate COP (*n* = 89)	Low COP (*n* = 89)
Patient characteristics				
Age, yr	67 (58–74)	70 (63–77)	69 (61–75)	59 (51–67)
Sex (female), *n* (%)	82 (30)	22 (25)	28 (31)	29 (33)
BMI, kg·m^−2^	29 ± 5	27 ± 4	29 ± 5	30 ± 6
Current smoker, *n* (%)	33 (12)	9 (10)	11 (12)	10 (11)
Diabetes mellitus, *n* (%)	71 (26)	28 (31)	23 (26)	15 (17)
COPD, *n* (%)	34 (12)	18 (20)	12 (13)	3 (3)
HF characteristics				
LVEF, %	34 (22–42)	33 (20–40)	33 (21–46)	35 (22–42)
Type of HF				
HFrEF, *n* (%)	199 (72)	66 (74)	60 (67)	63 (71)
HFmrEF, *n* (%)	45 (16)	13 (15)	13 (15)	19 (21)
HFpEF, *n* (%)	33 (12)	10 (11)	16 (18)	7 (8)
HF etiology				
Ischemic, *n* (%)	119 (43)	43 (49)	41 (47)	31 (35)
Idiopathic, *n* (%)	79 (30)	25 (28)	18 (20)	34 (38)
Other, *n* (%)	59 (21)	15 (17)	18 (20)	22 (25)
Valvular, *n* (%)	18 (6)	5 (6)	11 (13)	2 (2)
NYHA class				
Class 1, *n* (%)	28 (10)	6 (6)	8 (9)	13 (15)
Class 2, *n* (%)	143 (52)	47 (53)	44 (50)	47 (53)
Class 3, *n* (%)	103 (37)	36 (41)	34 (38)	29 (32)
Class 4, *n* (%)	3 (1)	0 (0)	3 (3)	0 (0)
Medical devices				
ICD, *n* (%)	122 (44)	39 (44)	37 (42)	41 (46)
Biventricular, *n* (%)	43 (35)	16 (41)	12 (32)	14 (41)
Pacemaker, *n* (%)	61 (22)	27 (30)	13 (15)	19 (21)
CRT, *n* (%)	52 (19)	17 (19)	13 (15)	20 (22)
Cardiac comorbidities				
Atrial tachycardia, *n* (%)	212 (77)	63 (71)	64 (72)	78 (88)
Atrial fibrillation, *n* (%)	140 (51)	48 (54)	40 (45)	44 (49)
PTCA, *n* (%)	72 (26)	26 (29)	25 (28)	18 (20)
CABG, *n* (%)	55 (20)	24 (27)	21 (24)	8 (9)
Heart transplantation, *n* (%)	0 (0)	0 (0)	0 (0)	0 (0)
Medication				
ACEI or ARB, *n* (%)	252 (91)	75 (84)	81 (91)	86 (97)
Aldosterone receptor antagonist, *n* (%)	179 (65)	58 (65)	56 (63)	57 (46)
β-blocker, *n* (%)	257 (93)	81 (91)	82 (92)	86 (97)
Diuretic, *n* (%)	250 (90)	81 (91)	85 (97)	74 (83)
Statin, *n* (%)	171 (62)	65 (73)	59 (66)	41 (46)
Laboratory values				
NT-proBNP, ng·L^−1^	813 (357–1820)	1430 (551–2870)	1035 (612–1770)	476 (235–981)
MDRD (mL·min^−1^ per 1.73 m)	58 (45–64)	54 (41–61)	55 (42–61)	61 (54–77)
Hemoglobin (mmol·L^−1^)	8.4 ± 1.0	8.3 ± 1.0	8.2 ± 1.0	8.7 ± 0.9
Na^+^ (mmol·L^−1^)	140 (138–141)	139 (137–142)	140 (138–141)	140 (138–141)
CR				
Type of CR				
3-month CR, *n* (%)	220 (79)	76 (85)	76 (85)	60 (67)
6-month CR, *n* (%)	57 (21)	13 (15)	13 (15)	29 (33)
80% adherence CR, *n* (%)	208 (76)	70 (79)	65 (73)	65 (73)
80% adherence 3-month CR, *n* (%)	161 (74)	59 (78)	56 (74)	39 (65)
80% adherence 6-month CR, *n* (%)	47 (83)	11 (85)	9 (69)	26 (90)

Data are presented as *n* (%), mean ± SD, or median (IQR).

ACEI, angiotensin-converting enzyme inhibitor; ARB, angiotensin-receptor blocker; CABG, coronary artery bypass grafting; CRT, cardiac resynchronization therapy; HFpEF, heart failure with preserved ejection fraction; e; HFmrEF, heart failure with midrange ejection fraction; ICD, implantable cardioverter–defibrillator; LVEF, left ventricular ejection fraction; MDRD, Modification of Diet in Renal Disease; PTCA, percutaneous transluminal coronary angioplasty.

### Characteristics associated with COP

**FIGURE 1:**
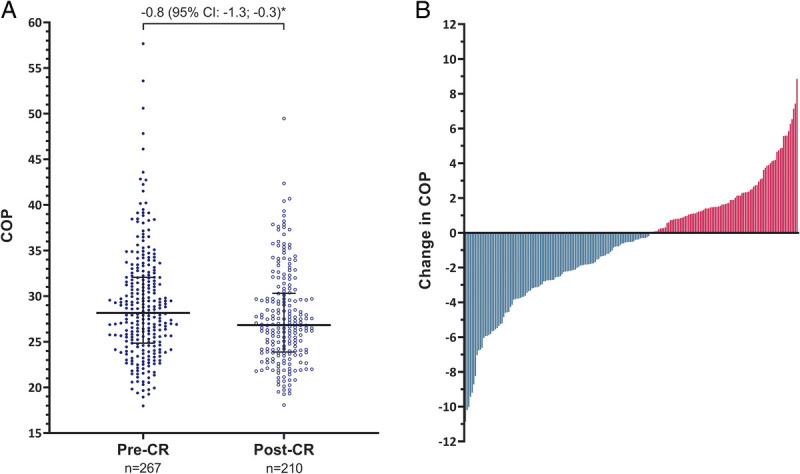
COP pre-CR (*solid blue dots*) and post-CR (*open blue dots*), with every dot representing one patient (A) and the change in COP (B). A, The COP improved from 28.2 (24.9–32.1) pre-CR to 26.8 (23.9–30.3) post-CR. A mixed model (*) adjusted for age, sex, type of HF, and NYHA class showed a decrease in COP from 0.8 (95% CI, −1.3 to −0.3). B, Absolute differences in COP from pre- to post-CR ranged from −10.9 to 8.9. Every *bar* represents one participant with an improved COP in green and a deteriorated COP in red.

The median COP value was 28.2 (24.9–32.1; Fig. 1) and was reached at 51% ± 15% of V̇O_2peak_. COP and V̇O_2peak_ were negatively correlated (see Supplemental Fig. 2, Supplemental Digital Content, Relation between the COP pre-CR and V̇O_2peak,_ http://links.lww.com/MSS/C862). Multivariable linear regression analysis showed that lower age, female sex, higher BMI, idiopathic HF compared with ischemic HF, the absence of a pacemaker or COPD, and lower NT-proBNP concentrations were associated with a lower COP (Table 2).

**TABLE 2:** Patient and disease characteristics associated with the COP (*n* = 267).

Variable	Univariable		Multivariable	
	*B* (95% CI)	*P*	*B* (95% CI)	*P*
Patient characteristics				
Age, yr	**0.19 (0.14 to 0.26)**	**<0.001**	**0.14 (0.08 to 0.20)**	**<0.001**
Female sex	**−1.73 (−3.31 to −0.15)**	**0.03**	**−1.74 (−3.15 to −0.33)**	**0.016**
BMI, kg·m^−2^	**−0.31 (−0.44 to −0.17)**	**<0.001**	**−0.25 (−0.38 to −0.12)**	**<0.001**
Current smoker (yes)	−0.28 (−2.59 to 2.03)	0.81		
Diabetes mellitus (yes)	1.46 (−0.22 to 3.14)	0.09	1.45 (−0.12 to 3.01)	0.07
COPD (yes)	**4.01 (1.85 to 6.17)**	**<0.001**	**3.16 (1.19 to 5.14)**	**0.002**
HF characteristics				
Type of HF				
HFrEF	REF			
HFmrEF	−1.02 (−2.99 to 0.95)	0.31		
HFpEF	−0.49 (−2.74 to 1.75)	0.66		
HF etiology				
Ischemic	REF		REF	
Idiopathic	**−2.59 (−4.31 to −0.86)**	**0.003**	**−1.92 (−3.46 to −0.37)**	**0.016**
Other	**−2.61 (−4.53 to −0.69)**	**0.008**	−1.54 (−3.25 to 0.18)	0.08
Valvular	−0.36 (−3.33 to 2.61)	0.81	−0.35 (−2.96 to 2.25)	0.79
NYHA class				
Class 1	−0.92 (−3.42 to 1.58)	0.47		
Class 2	REF			
Class 3	0.87 (−0.70 to 2.43)	0.28		
Class 4	−0.80 (−7.74 to 6.13)	0.89		
ICD (yes)	0.25 (−1.22 to 1.72)	0.74		
Pacemaker (yes)	**1.85 (0.11 to 3.59)**	**0.037**	**2.38 (0.82 to 3.95)**	**0.003**
CRT (yes)	−0.03 (−1.89 to 1.84)	0.98		
Atrial fibrillation (no)	−0.21 (−1.66 to 1.25)	0.78		
NT-proBNP, 1000 ng·L^−1^	**0.70 (0.41 to 0.99)**	**<0.001**	**0.52 (0.26 to 0.78)**	**<0.001**

Statistically significant findings (*P* < 0.05) are highlighted in bold text.

Data are presented as beta (*B*) with 95% CI. Multivariable linear regression analysis showed that a lower age, female sex, a higher BMI, no pacemaker implementation, no COPD, and low NT-pro BNP concentrations were associated with a lower COP.

CRT, cardiac resynchronization therapy; HFmrEF, heart failure with midrange ejection fraction; HFpEF, heart failure with preserved ejection fraction; ICD, implantable cardioverter–defibrillator.

### Impact of exercise training

An improvement in COP (−0.8; 95% CI, −1.3 to −0.3) was observed from pre-CR (28.2 (24.9–32.1)) to post-CR (26.8 (23.9–30.3); Fig. 1). These observations were reinforced by our sensitivity analysis including only patients with both a pre- and post-CR assessment (*n* = 200, COP pre-CR: 27.9 (24.6–31.5), change in COP: −0.7 (95% CI, −1.2 to −0.2)) and among patients without COPD (*n* = 243, COP pre-CR: 27.7 (24.4–31.5), change in COP: −0.8 (95% CI, −1.4 to −0.3)). Other cardiopulmonary variables, such as V̇O_2peak_, peak workload, and peak *V̇*_E_, were also improved from pre- to post-CR. Weight increased after CR, whereas NT-proBNP concentration, and systolic and diastolic blood pressure decreased (Table 3). Multivariable linear regression analysis revealed that only a higher COP pre-CR was associated with larger changes in COP. Patient or disease characteristics were not associated with exercise-induced changes in COP while adjusting for pre-CR COP (see Supplemental Table, Supplemental Digital Content, Patient and disease characteristics associated with exercise-based CR induced changes in COP, http://links.lww.com/MSS/C862).

**TABLE 3:** CPET outcomes and CVD risk factors at pre- and post-CR.

	Pre-CR (*n* = 277)	Missing Values, *n* (%)	Post-CR (*n* = 214)	Missing Values, *n* (%)	Change (95% CI)
Resting state					
Heart rate rest, bpm	74 (65–82)	0 (0)	—	214 (100)	—
Lactate rest, mmol·L^−1^	2.1 ± 0.7	3 (1)	2.0 ± 0.7	13 (6)	**−0.1 (−0.2 to −0.0)**
* V̇*_E_/V̇O_2_ rest	36.7 ± 7.1	118 (43)	36.0 ± 6.3	84 (39)	−0.3 (−1.4 to 0.7)
During the exercise protocol					
V̇O_2peak_, mL·kg^−1^·min^−1^	13.9 (11.0–17.5)	2 (1)	15.6 (12.5–19.5)	0 (0)	**0.9 (0.6 to 1.2)**
Peak heart rate, bpm	119 ± 27	2 (1)	122 ± 24	0 (0)	1 (−1 to 4)
Peak workload, W	89 (61–126)	2 (1)	103 (74–142)	0 (0)	**8 (5 to 11)**
Peak ventilation, mL·min^−1^	54 (42–72)	7 (3)	59 (46–77)	5 (2)	**3 (2 to 5)**
Peak RER	1.12 ± 0.11	5 (2)	1.13 ± 0.10	4 (2)	0.01 (−0.00 to 0.02)
Lactate end of protocol, mmol·L^−1^	3.6 (2.8–4.7)	12 (4)	3.8 (3.0–5.2)	15 (7)	0.2 (−0.0 to 0.4)
CVD risk factors					
Weight, kg	87.4 ± 18.1	0 (0)	88.8 ± 18.2	1 (1)	**1.0 (0.5 to 1.4)**
BMI, kg·m^−2^	29 ± 5	0 (0)	29 ± 5	1 (1)	0 (−0 to 1)
Systolic blood pressure, mm Hg	120 (107–137)	0 (0)	118 (107–133)	2 (1)	**−3 (−6 to −0)**
Diastolic blood pressure, mm Hg	75 ± 11	0 (0)	74 ± 12	2 (1)	**−2 (−3 to −0)**
NT-proBNP, ng·L^−1^	813 (357–1820)	0 (0)	626 (233–1390)	20 (9)	**−234 (−350 to −118)**

Statistically significant findings (*P* < 0.05) are highlighted in bold text.

Data are presented as *n* (%), mean ± SD, or median (IQR). The changes from pre- to post-CR are adjusted for age, sex, type of HF, and NYHA class and include the 95% CI. Cardiorespiratory (or aerobic) fitness (CRF) is represented by the variable V̇O_2peak_ (mL·kg^−1^·min^−1^).

RER, respiratory exchange ratio.

### COP and clinical outcomes

**FIGURE 2:**
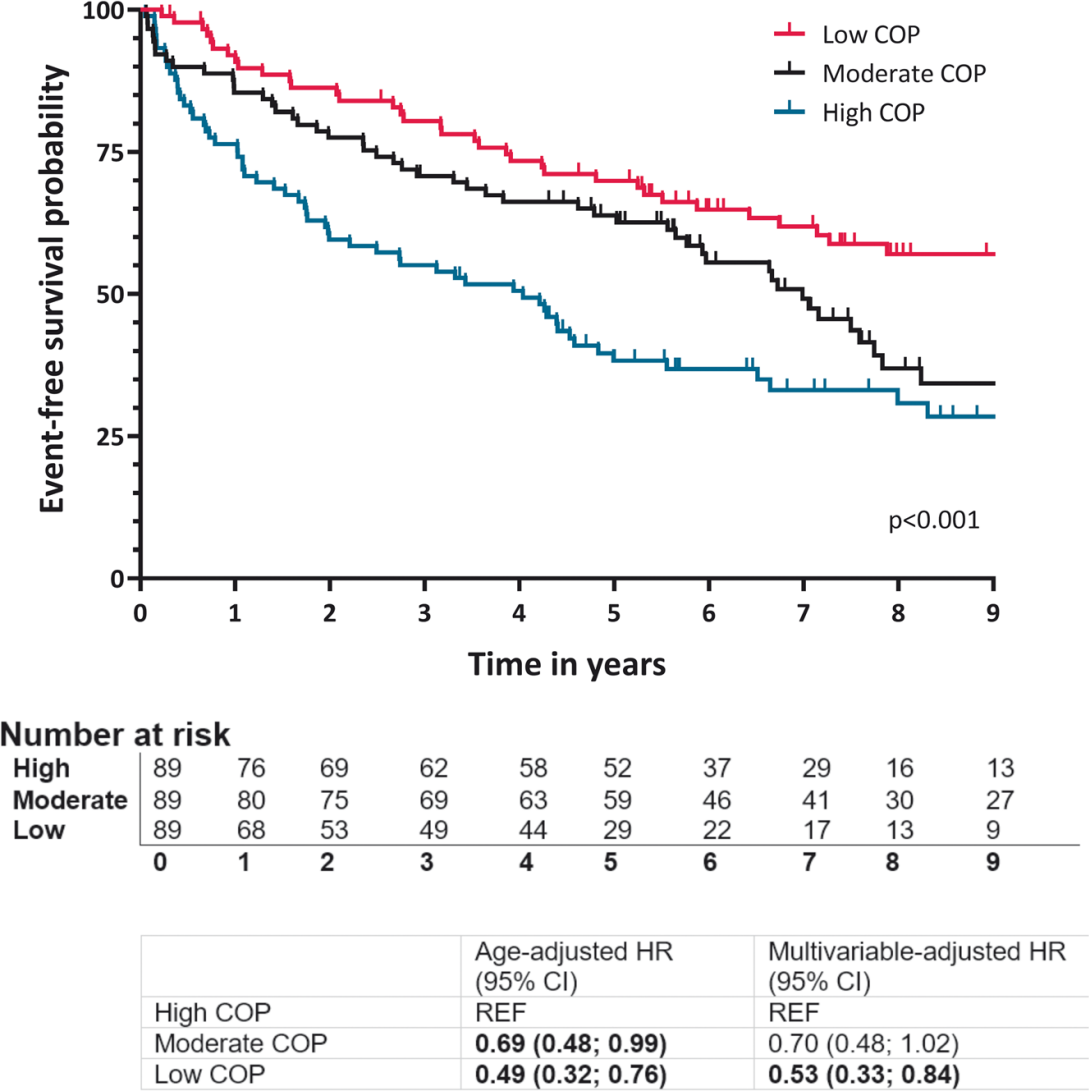
Kaplan–Meier curve of the time to clinical outcomes, and age-adjusted and multivariable-adjusted Cox proportional HR with 95% CI for the tertiles of COP assessed at pre-CR. Clinical outcomes were defined as all-cause mortality and CV-related unplanned hospitalization. HRs were adjusted for age, sex, type of HF, and NYHA class. The low COP group (18.0–26.0) had a 0.53 times risk (95% CI, 0.34 to 0.85) of clinical outcomes compared with the high COP group (30.7–57.7). HRs of the moderate COP group (26.0–30.7) did not differ from the high COP group.

One hundred sixty of 277 participants (58%) died or had a CV-related unplanned hospitalization during a median follow-up of 8.6 (6.0–10.7) yr. HF patients in the low COP tertile had significantly better event-free survival (HR_multivariable-adjusted_, 0.53; 95% CI, 0.33–0.84) compared with patients in the high COP tertile, whereas no differences in the event rate was found between the moderate and high COP tertiles after adjustment for confounders (HR_multivariable-adjusted_, 0.70; 95% CI, 0.48–1.02; Fig. 2). A sensitivity analysis with additional adjustment for V̇O_2peak_ showed an attenuated effect on event-free survival for the low COP tertile (0.66; 95% CI, 0.42–1.04) and the moderate COP tertile (0.79; 95% CI, 0.54–1.16) compared with the high COP tertile. Event-free survival did not differ between the tertiles of changes in COP (*P* = 0.13; see Supplemental Fig. 3, Supplemental Digital Content, Kaplan–Meier curve of the time to clinical outcomes, and age-adjusted and multivariable-adjusted cox proportional HR with 95% CI, http://links.lww.com/MSS/C862).

## DISCUSSION

This study performed a comprehensive assessment of the COP in patients with HF. We found that a lower age, female sex, higher BMI, idiopathic HF compared with ischemic HF, the absence of a pacemaker or the absence of COPD, and lower NT-proBNP concentrations were associated with a lower COP value. Furthermore, CR-based exercise training significantly reduced COP with 0.8 (95% CI, −1.3 to −0.3), which was independent of patient and disease characteristics. Finally, patients in the lowest COP tertile had a lower risk for all-cause mortality or CV-related unplanned hospitalization compared with patients in the highest COP tertile. Our findings suggest that the COP is a promising submaximal CPET variable for HF patients, given its association with classic CV risk factors, ability to improve after exercise training and its prognostic value for adverse clinical outcomes.

### Characteristics associated with COP

We found a median COP value of 28.2 in our cohort of HF patients. As expected, this value is higher compared with observations among healthier individuals from the general population (23.3–24.6) (7,9,10,13) but comparable to a previous study with HFrEF patients (29.6 ± 7.4) (14). More importantly, we found that classic CV risk factors were associated with the COP. General population studies previously revealed that a lower age (7,8), male sex (7,10), and a lower BMI (13) were associated with a lower COP. Our findings reinforce the impact of age, but not for BMI or sex. The link between a higher BMI and better clinical outcomes is previously described in patients with HF and might be a result of a structural bias. Although sarcopenic HF patients tend to have a poorer clinical prognosis, those patients with HF caused by a higher BMI or obesity might have a better event-free survival (15). This may also apply to the COP and other ventilatory efficiency variables (16). We cannot explain the association of sex on COP values, as a previous study found a poorer ventilatory efficiency in female HF patients (17). Finally, we identified NT-proBNP concentrations, HF etiology, and the presence of a pacemaker or COPD as novel correlates of COP. These findings, together with the normative data, provide important information on how to interpret the COP in patients with HF.

### Impact of exercise training

To our knowledge, this is the first study to report that the COP can improve after exercise training in patients with HF. This observation aligns with previous studies that showed that other measures of ventilatory efficiency, such as the *V̇*_E_/V̇CO_2_ slope, can be improved by exercise training (18). Nevertheless, the average COP improvement was modest in our study (pre-CR: 28.2 (24.9–32.1); change after CR: −0.8 (95% CI, −1.3 to −0.3}) compared with previously reported *V̇*_E_/V̇CO_2_ slope improvements (6%–23%) in heart patients (18). A potential explanation for the discrepant findings may be that persons with a lower, more normal COP are less able to change their COP after CR, as baseline COP and changes in COP were negatively associated. Taken together, the COP is sensitive to changes after CR-based exercise training, especially in those with abnormally high values, which may be used to evaluate improvements in cardiorespiratory function.

### COP and clinical outcomes

In line with our hypothesis, we found that a low COP was associated with a lower risk for adverse clinical outcomes during long-term follow-up. Our data support previous findings as a high COP was associated with all-cause mortality in community-dwelling adults (8) and a higher risk of heart transplantation or mortality among HFrEF patients after 1-yr follow-up (14). Although the sensitivity analysis with additional adjustment for V̇O_2peak_ showed attenuated hazard ratios, the change in effect size was small. These results emphasize that COP and V̇O_2peak_ are different measures of cardiopulmonary function and future research should examine the joint value of COP and V̇O_2peak_ and its potential to replace V̇O_2peak_ when maximal exercise testing is less convenient in HF. These collective findings underline the robustness of COP as a prognostic variable for event-free survival across various cohorts, including HF patients.

### Clinical relevance

The COP is a promising variable for risk stratification among chronic HF patients given its association with CV risk factors, modifiability after exercise training, and prognostic value for mortality and unplanned hospitalization. A key advantage of the COP is that it can be assessed during a submaximal exercise test with expired gas analysis. Indeed, we found that COP is reached at 51% ± 15% of V̇O_2peak_, and this point may coincide with the first ventilatory threshold (19). Submaximal exercise tests are cheaper, easier to perform, and less burdensome for patients compared with maximal exercise tests (20). Another benefit is the observer-free error of the COP, as it is determined by taking the lowest minute value (7). This contrasts with *V̇*_E_/V̇CO_2_ slope or determining anaerobic thresholds, as there are different definitions and methodological strategies for these variables (21), which could affect the risk of bias. Taken together, the accessible assessment of COP in combination with its clinical value may increase the utility of CRF testing in HF care programs.

### Strengths and limitations

The strength of the study includes our comprehensive assessment of the COP in HF patients. However, there were also some limitations. First, our study sample mainly consisted of HFrEF patients (72%), but the linear regression analysis showed no impact of type of HF on COP. Second, COP data at pre- and post-CR were not available in the full cohort (80/280 patients; 28%), but our complete case sensitivity analysis (*n* = 200) confirmed our main outcomes. Finally, our study sample included 34 COPD patients with HF. As COP is a variable that compromises both the cardio- and pulmonary system, ventilatory problems due to COPD might influence the effect on COP. However, a sensitivity analysis showed that the change in COP was comparable in our subpopulation without COPD, and we adjusted for COPD in our Cox regression analysis.

## CONCLUSIONS

Classic CV risk factors are associated with a higher, more unfavorable, COP value. CR-based exercise training significantly reduces COP values, whereas a lower COP is associated with a better clinical prognosis. As COP can be easily quantified during a submaximal incremental exercise test, this may offer novel risk stratification possibilities for HF care programs.
